# Pneumonia Caused by *Shigella sonnei* in Man Returned from India

**DOI:** 10.3201/eid1511.090126

**Published:** 2009-11

**Authors:** Fabiola Mancini, Antonella Carniato, Alessandra Ciervo

**Affiliations:** National Public Health Institute, Rome, Italy (F. Mancini, A. Ciervo); Presidio Ospedaliero, Treviso, Italy (A. Carniato)

**Keywords:** Pneumonia, *Shigella sonnei*, traveler, India, PCR, sequencing, letter

**To the Editor:** Shigellosis is a cause of infectious dysentery frequently occurring in developing countries yet usually associated with diarrhea in travelers from regions where *Shigella* infections are endemic. *Shigella* spp. are usually spread directly from person to person by the fecal–oral route or indirectly by fecal contamination of food or water with ingestion of feces contaminated food or water (*1*). Aside from clinical intestinal manifestations, shigellosis causes a wide variety of extraintestinal signs, such as bacteremia or neurologic manifestations (*2*).

Pneumonia is an atypical but potential complication of shigellosis. In developing countries, *Shigella sonnei* and *S. flexneri* infections were reported to cause acute pneumonia in malnourished infants and, in these cases, were associated with severe prognosis and a death rate of 14% (*3*).

We describe a case of severe pneumonia caused by *S. sonnei* that developed in a man from Italy who had traveled to India. This is an atypical case of shigellosis occurring in an immunocompetent person, generally healthy and without any underlying severe predisposing condition.

A 69-year-old white man was admitted to the emergency unit of the Presidio Ospedaliero, Department of Infectious Diseases, Treviso, Italy, on February 24, 2008, with severe dyspnea and a cough producing purulent sputum. He had traveled to India and had visited urban and rural areas over a 15-day period. He returned home 7 days before hospital admission. During his travel, the patient reported episodes of vomiting and moderate diarrhea without fever. These signs were resolved 4 days before his return to Italy. Initial examination showed he had a temperature of 37°C and an oxygen saturation of 88% in room air. Arterial blood gas levels were pH 7.42, partial pressure of oxygen in arterial blood 42 mm Hg, and partial pressure of carbon dioxide 35 mm Hg. Because of his progressive respiratory failure, he was transferred to the intensive care unit. Relevant laboratory tests were performed, and abnormal values of erythrocyte sedimentation rate 85 mm/h, C-reactive protein 105 mg/L, hemoglobin 10.2 g/dL, and neutrophilia were found.

A chest radiograph showed diffuse pneumonia with infiltrates. A computed tomography scan of the thorax showed nodular lesions and cavity formations. No neurologic or abdominal abnormalities were found, and peristalsis was within normal limits.

Sputum and bronchial alveolar lavage (BAL) smears showed gram-negative microorganisms. Melioidosis was suspected because the man had traveled to a known melioidosis-endemic area. In view of this information, blood, sputum, and BAL samples were collected, and the patient was immediately given empirical antimicrobial drug therapy with amoxicillin/clavulanic acid, plus meropenem and norfloxacin. Given the absence of gastrointestinal symptoms and because shigellosis was not suspected, stool samples were not obtained. Specimens were sent to the Istituto Superiore di Sanità, Infectious Diseases Department for bacteriologic examination. Blood cultures were negative; gram-negative rods were recovered from the sputum and BAL smears. The microorganisms were identified as *S. sonnei* by the API 20E strip (bioMerieux Italia, Florence, Italy). The bacteria agglutinated in *Shigella* group D antiserum but failed to agglutinate in *Shigella* groups A, B, and C antisera (Becton Dickinson Diagnostic Systems Italia, Milan, Italy). To further confirm bacterial identification, we amplified the full length of 16S rRNA nucleotide sequence by using the universal primers for eubacteria, 16S rRNAs (27f 5′-GAGAGTTTGATCCTGGCTCAG-3′ and 1495r 5′-CTACGGCTACCTTGTTACGA-3′) and sequenced the PCR product. The sequence obtained was compared by using the BLAST search tool (www.ncbi.n/m.nib.gov/BLAST), which showed 100% identity with the 16S rRNA *S. sonnei* strain AU65 sequence GenBank accession no. EF032687). Antimicrobial drug susceptibility of the isolate was determined for 26 agents by the disk-diffusion method in Mueller Hinton agar, according to the Clinical and Laboratory Standards Institute. The isolate was susceptible to amikacin 30 µg/mL, ceftazidime 30 µg/mL, ceftriaxone 30 µg/mL, meropenem 10 µg/mL, sulfisoxazole 0.25 µg/mL, sulfonamides 300 µg/mL, and triple sulfa 23.75/1.25 µg/mL; intermediate to cefotaxime 30 µg/mL, gentamicin 10 µg/mL, kanamycin 30 µg/mL, and tobramycin 10 µg/mL; resistant to amoxicillin 25 µg/mL, amoxicillin/clavulanic acid 20/10 µg/mL, ampicillin 10 µg/mL, ampicillin-sulbactam 20 µg/mL, cefoxitin 30 µg/mL, chloramphenicol 30 µg/mL, ciprofloxacin 5 µg/mL, clarithromycin 15 µg/mL erythromycin 15 µg/mL, nalidixic acid 30 µg/mL, norfloxacin 10 µg/mL, streptomycin 10 µg/mL, tetracycline 30 µg/mL and trimethoprim 5 µg/mL.

The patient was discharged from hospital after 40 days. At follow-up 6 months later, his general health status was good, and a chest radiograph showed no abnormalities.

Extraintestinal signs associated with *S. sonnei* infections are generally reported as secondary manifestations of dysentery. In particular, bacteremia is reported as a gastrointestinal complication in infants in developing countries (*4*) or in immunocompromised adults (*5*); pneumonia associated with *S. sonnei* is more rare but possible and has been described in malnourished children (*4**,**6*), in human immunodeficiency virus–infected patients (*7*), and in patients with chronic diseases (*8*–*10*). Generally, in these cases, pneumonia is associated with bacteremia.

This reported case of severe pneumonia related to *S. sonnei* is unusual in a healthy patient with self-limiting dysentery whose symptoms and clinical conditions were not suggestive of bacteremia. Vomiting and aspiration of mixed mouth flora containing *Shigella* spp. could be a possible cause of pneumonia in this patient. However, the hematogenous route cannot be excluded. A potential explanation of the severe illness could be that in healthy elderly people the immune system functions are less vigorous and thus more susceptible to infections. Nevertheless, the acute episode in this patient was effectively treated by a combination of meropenem, norfloxacin, and amoxicillin/clavulanic acid, although the bacterium is resistant to the latter 2 drugs.

This case report should be of particular interest for clinicians because it describes an atypical case of extraintestinal shigellosis and an example of misdiagnosis of melioidosis. Clinicians should be alert for pneumonia associated to *Shigella* spp. or *Burkholderia pseudomallei,* specifically in healthy people who have traveled to areas to which these pathogens are endemic.

## References

[1] Centers for Disease Control and Prevention. *Shigella*. Annual summary. Atlanta (GA): The Centers; 2002.

[2] Ashkenazi S. *Shigella* infections in children: new insights. Semin Pediatr Infect Dis. 2004;15:246–52. 10.1053/j.spid.2004.07.005 15494948

[3] Bennish ML. Potentially lethal complications of shigellosis. Rev Infect Dis. 1991;13:S319–24. 2047657 10.1093/clinids/13.supplement_4.s319

[4] Dutta P, Mitra U, Rasaily R, Bhattacharya SK, Bhattacharya MK. Assessing the cause of pediatric in-patients diarrheal deaths: an analysis of hospital records. Indian Pediatr. 1995;32:313–21. 8613286

[5] Mandell W, Neu H. *Shigella* bacteremia in adults. JAMA. 1986;255:3116–7. 10.1001/jama.255.22.3116 3517402

[6] Garanin A. Isolation of *Shigella sonnei* from the lung of a child who died of acute dysentery [in Russian]. Pediatriia. 1970;49:81–2. 4915114

[7] Miller RF, Symeonidou C, Shaw PJ. Pneumonia complicating *Shigella sonnei* dysentery in an HIV infected adult male. Int J STD AIDS. 2005;16:763–5. 10.1258/095646205774763243 16303074

[8] Hawkins C, Taiwo B, Bolon M, Julka K, Adewole A, Stosor V. *Shigella sonnei* bacteremia: two adult cases and a review of the literature. Scand J Infect Dis. 2007;39:170–3. 10.1080/00365540600786580 17366038

[9] Margolin L, Engelhard D. Bilateral pneumonia associated with *Shigella sonnei* dysentery. Am J Infect Control. 2003;31:445–6. 10.1067/mic./2003.69 14650419

[10] Raffensperger EC. Combined bacillary and amebic ulcerative colitis associated with atypical pneumonitis and *Shigella*-positive sputum. Am J Med. 1956;20:964–7. 10.1016/0002-9343(56)90263-7 13326908

